# Wearable-Sensor-Based Detection and Prediction of Freezing of Gait in Parkinson’s Disease: A Review

**DOI:** 10.3390/s19235141

**Published:** 2019-11-24

**Authors:** Scott Pardoel, Jonathan Kofman, Julie Nantel, Edward D. Lemaire

**Affiliations:** 1 Department of Systems Design Engineering, University of Waterloo, Waterloo, ON N2L 3G1, Canada; spardoel@uwaterloo.ca; 2 School of Human Kinetics, Faculty of Health Sciences, University of Ottawa, Ottawa, ON K1N 6N5, Canada; jnantel@uottawa.ca; 3 Faculty of Medicine, University of Ottawa, Ottawa Hospital Research Institute, Ottawa, ON K1H 8M2, Canada; elemaire@ohri.ca

**Keywords:** Parkinson’s disease, freezing of gait, wearable sensors, detection, prediction, machine learning

## Abstract

Freezing of gait (FOG) is a serious gait disturbance, common in mid- and late-stage Parkinson’s disease, that affects mobility and increases fall risk. Wearable sensors have been used to detect and predict FOG with the ultimate aim of preventing freezes or reducing their effect using gait monitoring and assistive devices. This review presents and assesses the state of the art of FOG detection and prediction using wearable sensors, with the intention of providing guidance on current knowledge, and identifying knowledge gaps that need to be filled and challenges to be considered in future studies. This review searched the Scopus, PubMed, and Web of Science databases to identify studies that used wearable sensors to detect or predict FOG episodes in Parkinson’s disease. Following screening, 74 publications were included, comprising 68 publications detecting FOG, seven predicting FOG, and one in both categories. Details were extracted regarding participants, walking task, sensor type and body location, detection or prediction approach, feature extraction and selection, classification method, and detection and prediction performance. The results showed that increasingly complex machine-learning algorithms combined with diverse feature sets improved FOG detection. The lack of large FOG datasets and highly person-specific FOG manifestation were common challenges. Transfer learning and semi-supervised learning were promising for FOG detection and prediction since they provided person-specific tuning while preserving model generalization.

## 1. Introduction

Parkinson’s disease (PD) is a progressive neurodegenerative condition that presents numerous life-altering symptoms, including the characteristic upper-limb trembling [1]. In moderate to advanced PD, locomotion can deteriorate into a flexed upper body posture with small shuffling steps, an anteriorly-shifted centre of mass, decreased walking speed, poor balance, increased gait variability, and freezing of gait (FOG) [2,3,4,5,6].

A FOG episode is a complex and highly-variable phenomenon defined as a “brief, episodic absence or marked reduction of forward progression of the feet despite the intention to walk” [7]. Freezing is often described as the sensation of having one’s feet glued to the floor with an inability to initiate the next step, and becomes increasing common as PD progresses [2,8]. Although typically lasting only a few seconds [9], freezes can lead to falls [10,11,12]. Since FOG can occur multiple times a day, most commonly between doses when medication is wearing off [11,13], FOG related fall risk is an ever-present concern. Fall-related injury, reduced mobility, fear of future falls, and decreased independence are all linked to FOG and can contribute to a reduced quality of life [14,15,16,17,18]. 

FOG occurrences are difficult to anticipate and may not manifest during clinic visits [3]. Therefore, assessing and adjusting FOG treatments can be challenging for medical professionals. In-home monitoring and automatic freeze-detection systems have been developed [19,20] and used to objectively track freezes over extended periods; however, these systems do not prevent or reduce freezing occurrences. Cueing devices that provide an external stimulus have emerged for preventing imminent or overcoming occurring FOG episodes [21,22]. Continuous stimuli include auditory (e.g., a rhythmic tone), visual (e.g., lines projected on the floor in front of the person), or tactile (e.g., a vibrating device on the skin). As an alternative to continuous cueing, some cueing systems detect an occurring freeze and provide a cue to help the person resume normal walking [23,24,25,26]. If FOG could be predicted, a cue could be provided before the event to prevent the freeze from occurring [27,28].

Accurate and automatic FOG detection and prediction are essential for long-term symptom monitoring or preemptive mitigation via cueing. Wearable sensors are vital for FOG detection and prediction systems, to ensure unrestricted daily use in a person’s chosen environment. The complexity of FOG symptoms and its highly-variable manifestations have led to the creation of systems with numerous sensors on various body parts and a wide array of FOG detection methods, ranging from simple thresholds to machine-learning approaches. While good results have been reported, automatic and reliable FOG detection and prediction is far from resolved. Previous review of the state of the art technology in this field has shown the widespread use and effectiveness of wearable sensors in FOG detection [26]. To help guide further research, the current paper presents and up-to-date review of the state of the art of FOG prediction, and provides further details on the study populations, classification methods, and features used. The current state of the art is also assessed to highlight current challenges and limitations in FOG detection and prediction using wearable sensors. The outcomes from this review provide guidance on current knowledge and identify knowledge gaps that need to be filled to advance wearable-sensor-based assistive technologies that can improve the lives of people with PD.

## 2. Materials and Methods

A literature review was performed by searching the Scopus, PubMed, and Web of Science databases. Keywords included “sensor” or “device” or “wearable”, “Parkinson”, “freeze” or “freezing”, “detect”, or “predict”. The final search was performed on April 16, 2019. Results were curated using the Mendeley Desktop software (v. 1.19.4) (Elsevier, Amsterdam, the Netherlands).

Duplicates were removed and the results were pooled for screening, using article title, abstract, and keywords to determine relevance. Following screening, the remaining documents were reviewed in full. 

Eligibility for analysis was based on:

1. Use wearable sensor data as input (direct from sensor or wearable sensor datasets).
2. Involve people with PD, or data from people with PD, who experience FOG.
3. Primary goal of detecting or predicting FOG. Articles were not included if they examined cueing using a FOG detection method developed in previous research and reported in another article, or if they only classified individuals as freezers or non-freezers, rather than detecting freezing episodes.

Articles were excluded if they were not published in English, if they were not full texts (abstract only publications were excluded), or if they lacked adequate descriptions and explanations of the detection or prediction methods (i.e., training and testing methods not described, important variables not defined, results not presented).

Eligible articles were used to extract, where available, the following characteristics: population, data collection location and summary, sensor type and location, FOG detection and prediction method (i.e., classifier or machine-learning algorithm), features, whether feature extraction and selection were used, classification performance, and evaluation in real-time. 

Article characteristics included:

- Population: The number of participants in the study, i.e.: healthy controls (HC), people with FOG symptoms (FOG-PD), people with no FOG symptoms (NFOG-PD), and FOG symptom status unknown or not reported (UFOG-PD); the number of PD participants who froze during data collection, medication state during data collection (ON or OFF), number of FOG episodes.
- Data collection location and summary: Whether data collection was performed in a laboratory setting or in the participant’s home. Summary of walking tasks performed.
- Sensor type and location: The type and number of sensors used, sensor location on the body.
- FOG detection method: Methods used to detect and predict FOG, i.e., general approach (e.g., machine-learning model), model training method (person-specific: trained using data from a single person; or person-independent: trained using data from multiple people and not customized for an individual), whether the data was windowed, window length, and extent of detection (i.e., detection performed on each data point, window, or FOG event, etc.). Where multiple methods were attempted, the method with the best performance or research focus was reported.
- Feature extraction and feature selection: Features are variables calculated from sensor data. Feature selection uses feature ranking, filtering, or other techniques to produce an appropriate feature subset with fewer redundant features. Reporting features that performed best in FOG detection or comparing detection performance of different features after model testing was not considered as feature selection.
- Classifier performance: Sensitivity, specificity, other performance metrics reported.
- Real-time: Reporting the detection of a FOG episode as it occurs. In this review, real-time refers to detection using a live wearable-sensor data stream.

Feature analysis included:

- Feature Name: Feature name or a short description if not named in the cited article.
- Sensor Type: The type of sensor to calculate the feature: accelerometer (Acc), gyroscope (Gyro), force sensitive resistor (FSR), electromyography (EMG), electroencephalogram (EEG), galvanic skin response (GSR), goniometer, telemeter, or camera-based motion capture (CBMC) (included if used with wearable-sensor).
- Sensor Location: Body location where the sensor was placed.
- Feature Description: Brief explanation of the feature.
- Source: Articles that used the feature as input for FOG detection or prediction.

## 3. Results

**Figure 1:**
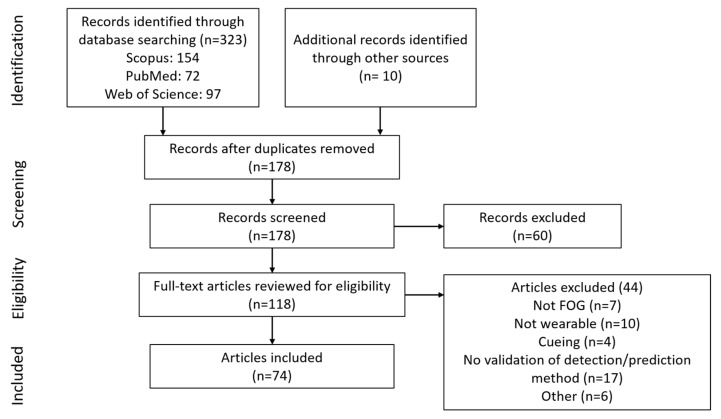
Diagram of article selection process.

The initial search provided 323 documents. An additional 10 articles that did not appear in the search but were referenced by other articles were included, resulting in 333 articles. After removing duplicates, 178 documents were available (Figure 1). Following screening and eligibility assessment, 74 articles were included in the review: 68 on FOG detection, seven on FOG prediction, and one article in both categories. 

Study characteristics related to population, data collection location and summary, sensor type and location, FOG detection method, feature extraction and selection, classifier performance, and whether analysis was performed in real time are presented in Table 1. Features extracted from wearable sensor data are presented in Table 2. Table 3 presents a summary of the top machine-learning methods from studies that compared different machine-learning classifiers for FOG detection using wearable sensors. 

## 4. Discussion

### 4.1. FOG Detection 

FOG detection methods vary in complexity, with the simplest models directly comparing wearable sensor variables to thresholds [29,41,44,46,53,63,73,123]. Threshold methods tended to have poorer detection performance but faster processing time, making them potentially useful in real-time systems [24,70,77,79,92,93]. To improve classification performance, features that can better differentiate between FOG and typical PD gait have been used, such as Fourier transforms [29,34,35,41,44,53,65,69,78], wavelet transforms [51,56,63,71,79,83,91,92,93,96], k-index [59,60,61,62,72,73], freezing of gait criterion (FOGC) [46], freezing of gait detection on glasses (FOGDOG) [70], R-index [94], and the widely-used freeze index [29]. 

To further improve FOG detection performance, multiple features and machine-learning (ML) techniques have been used, such as neural networks [36,38,55,66,76,80,85,86,88,89,91], decision trees [25,39,42,45,52,54,58,85], random forests, [39,42,43] naïve Bayes [42,43], nearest neighbor [42], and support vector machines [64,71,74,75,81,83,86]. In addition, anomaly detection [20] and unsupervised machine learning have been attempted [87], but not extensively explored.

The best performing classifiers for FOG detection were convolutional neural networks, support vector machines, random forest, and AdaBoosted decision trees (Table 3).

#### 4.1.1. Decision Trees

Decision trees are a series of binary selections that form branches resembling a tree structure. More complex decision trees can improve performance. For example, random forest classifiers use multiple decision trees, where the final decision is the majority vote of the individual trees. Boosting can also improve performance. AdaBoosting (adaptive boosting) repeatedly retrains the classifier, placing increasing importance on incorrectly classified training examples [124,125]. LogitBoosting (logistic boosting) [126], RUSBoosting [127], and RobustBoosting [128] are extensions of AdaBoosting that can further improve performance [85]. Decision trees for FOG detection included ensembles of trees and boosting techniques [42,43,85], with performance results ranging from 66.25% to 98.35% for sensitivity and 66.00% to 99.72% for specificity [25,39,42,43,45,52,54,58,85].

#### 4.1.2. Support Vector Machines (SVM)

Support vector machines are binary (two class) classifiers that trace a plane to separate data points from each class. New data points are then classified based on their side of the plane. If data points are not easily separable, a kernel can transform the data into a dimension that is linearly separable [125]. SVM for FOG detection achieved 74.7%–99.73% sensitivity and 79.0%–100% specificity [64,71,74,75,81,83,86].

#### 4.1.3. Neural Networks

Neural networks (NN) are made up of interconnected layers of nodes inspired by the structure of neurons in the brain [129]. NN have been frequently used in FOG detection and prediction studies. For FOG detection, model performance achieved 72.2%–99.83% sensitivity and 48.4%–99.96% specificity [36,38,55,66,76,80,85,86,88,89,91]. Neural networks for FOG prediction tended to perform slightly worse, up to 86% sensitivity, 80.25% specificity, and 89% precision [96,97,99].

Different NN subtypes have been used in FOG detection and prediction, such as convolutional [85,90] and recurrent [97,100] NN. Convolutional neural networks (CNN) have become popular in numerous applications, including medical image analysis, in part due their ability to recognize local patterns within images and because feature selection prior to implementation is not required [130,131]. CNN performed well for FOG detection [85], achieving 91.9% sensitivity and 89.5% specificity. Recurrent NN have recently been used for FOG prediction due to their applicability to time-series data [97,100]. Recurrent neural networks (RNN) utilize previous data in addition to current inputs during classification [132], thus giving the network “memory” to help recognize sequences [133]. A long short-term memory network (LSTM), a type of RNN, was used for FOG prediction [100], achieving over 90% accuracy when predicting FOG 5 s in advance. 

#### 4.1.4. Unsupervised and Semi-Supervised Models

Since freezing manifests differently for each person, person-specific models outperformed person-independent models [42,58,74,86] (with some exceptions as in [53]). However, in practice, it is difficult to obtain enough data to develop a model for an individual. To address this small dataset problem, unsupervised learning has been attempted. These methods do not rely on experts labelling FOG episodes. Instead, clustering techniques are used to define the classes [87], or an anomaly detection approach is used to define the normal class and then identify abnormalities (such as FOG) that do not conform to that class [45,90]. Unsupervised FOG detection approaches are appealing since they do not require data labelling; however, few studies have used unsupervised FOG detection, and unsupervised models performance has been worse than supervised models [90].

Recently, transfer learning, which uses a previously-trained network as a base and adapts the model to a new task [100], and semi-supervised learning, which uses both labeled and unlabeled data during training [69,88,89], have been used to create partly personalized FOG detection methods without large amounts of data. In [100], transfer learning trained a neural network using group data before adding an additional network layer that was trained using an individual’s data. Semi-supervised learning methods [69,88,89] use labeled data to train a base classifier before updating in an unsupervised manner. This reduces the need for labeled data and preserves the generalization ability from a multiple person data set, while allowing person-specific tuning. Semi-supervised learning theoretically combines the advantages of both supervised and unsupervised learning. When applied to FOG detection, performance achieved 89.2%–95.9% sensitivity [69,88,89] and 93.1%–95.6% specificity [69,88,89]. Although the methods are promising, due to a current shortage of studies, the value of these methods for FOG detection remains unclear.

#### 4.1.5. Limitations and Challenges of FOG Detection

FOG detection and prediction is affected by the participant’s medication state (ON and OFF), with substantial effects on motor control, gait patterns, and physical abilities. Freezing occurs more frequently in the OFF state than the ON state. In the OFF state, smaller shuffling steps are common, whereas in the ON state, many people can walk fairly normally. A machine-learning model trained during a person’s optimal medication state may perform worse if the medication wears off and their unassisted gait changes. Given that medication is needed in PD management, medication state is crucial contextual information for FOG detection and prediction research.

With machine-learning algorithms becoming more prevalent, larger FOG detection and prediction datasets are needed for model development. FOG studies ranged from 1 to 32 participants, with most studies having more than 10 participants. Studies involving few participants may not adequately validate a FOG detection method, especially when machine-learning algorithms are involved. Data augmentation techniques [85] or additional testing with more participants are required. On the other hand, large participant pools may not guarantee unbiased datasets since some participants freeze many times during data collection, while others may not freeze at all. For example, in [48], only 6 of 20 participants froze during data collection, which may lead to person-biased models that over-represent the few individuals with FOG data. Difficulty in participant recruitment and FOG unpredictability are therefore challenges that may limit the availability and quality of training data. 

Following data collection, FOG episodes are typically visually identified and labelled. Visual FOG identification is currently the gold standard. These labels are ground truth for detection method validation. Even though FOG is a well-defined clinical phenomenon [7], the criteria for defining the beginning and end of FOG episodes [24,25,98] was not defined in some articles. Differing FOG definitions make comparison between studies problematic. Published datasets can provide consistent ground truth FOG labelling. The Daphnet [24] (10 participants) and CuPiD [101] (18 participants) datasets provide consistent input but fewer than 250 FOG episodes; thus, dataset size may be an issue for machine learning, especially if deep learning is used [85].

When evaluating a classification system, ideally, different data are used for training and testing, as in [25,38,51,55,56,64,66,67,85,96,97,99,100], in order to prevent model performance overestimation that can occur when the model is evaluated using data used in model training. Cross-validation is often used when the dataset size is limited, as done in [24,31,32,33,39,42,43,45,52,54,58,74,75,81,86,87,88,89,90,98]. For FOG research, leave-one-person-out cross-validation was the most common. In this method, model training used data from all but one participant, model testing used data from the remaining participant, the process was repeated for each participant, and the performance results were averaged. Other studies, often more preliminary in nature, used ad hoc optimization to tune parameters and set thresholds [34,44,48,59,60,61,62,63,95]. This approach, although useful for initial system assessment, is not a good indicator of classifier performance, and should be followed by a more robust evaluation scheme, such as cross-validation.

Feature calculation from wearable sensor data is typically done using data windows. Window lengths ranged from 0.2 to 32 s [36,48,92,93], with the most common window length being 1 s. Long windows with many sample points are desirable for calculating frequency-based features involving the discrete Fourier transform, since the number of sample points in the input signal will determine the output frequency bin resolution. However, long windows decrease the temporal resolution and do not permit distinguishing short events within the window. In addition, long windows with many data points may be slower to process and may introduce unwanted lags between data acquisition and classification for detection or prediction. Studies comparing multiple window lengths found that, in general, 1–4 s windows are preferable [42,44,48,57,63,64].

FOG detection studies used different performance metrics. For example, a FOG detection system used to trigger a real-time cue during walking might emphasize freeze onset detection. This detection system might attempt to classify every data point or window as FOG or no freeze, and be evaluated using the number of correctly classified instances [24,31,32,33]. In contrast, a long-term monitoring system may treat each freeze occurrence as a binary event and evaluate whether the FOG event was successfully detected [74,75]. Experimental procedures and underlying definitions, such as ignoring FOG shorter than 3 s [43] or calculating specificity with data from participants without FOG [64], also varied between studies. Differences in evaluation metrics and procedures make FOG detection method comparisons more difficult. 

To help compare future FOG detection and prediction studies, researchers should include study population details; including, sex, PD severity, number of participants, the number and duration of FOG episodes (ideally for each person), and medication state during testing. Methodologically, the FOG labelling criterion, detailed detection method, validation method, and basis upon which the performance evaluation metrics are calculated should be clearly stated. 

### 4.2. FOG Prediction

The FOG prediction studies varied in approach and performance, with most being somewhat preliminary and focusing less on performance and more on understanding the intricacies of FOG prediction. In addition to FOG detection study considerations (e.g., dataset size, medication state, FOG definitions, contextual or study-specific performance metric definitions), FOG prediction studies must define the pre-FOG class using data before freeze onset. FOG prediction is typically done by training a machine-learning model to recognize data from the pre-FOG class. Six of the seven FOG prediction studies selected a pre-FOG segment duration that ranged from 1 s [45,96,100] to 6 s [45]. Since the transition from walking to FOG is subtle, labelling the start of pre-FOG from visual observation is difficult. Instead, a FOG episode is visually identified, and data prior to the FOG are selected using a single fixed duration. Three studies used a 5 s period [96,97,99]; one study used a 2 s period [98]; one used 1,3 and 5 s periods [100]; and one used 1–6 s periods, in 1 s increments [45]. The seventh study [95] used an assumed 3 s period before FOG for feature selection; then, a person-specific, multivariate Gaussian-distribution-based anomaly-detection model was created and manually tuned for each participant. 

Optimal pre-FOG segment duration is difficult to determine. If the pre-FOG segment is assumed to be a linear degradation of gait leading to FOG (threshold theory [134]), data closest to the freeze would resemble FOG, and data farther from the freeze would resemble typical PD walking. For a two-class classifier (pre-FOG, typical PD walking), short pre-FOG segments are preferred, since data are closer to FOG onset and likely more distinct from typical walking [100]. 

A short pre-FOG segment may not be ideal when using a three-class classifier consisting of typical PD walking, pre-FOG, and FOG classes as in [45], which found that very short pre-FOG segments made it difficult to distinguish between the pre-FOG and FOG classes. Longer pre-FOG segments improved pre-FOG classification but greatly reduced FOG and typical walking classification accuracy. The best performing pre-FOG segment duration differed across participants, and likely between individual FOG episodes for the same person [45]. The observation that a single pre-FOG duration is inadequate is also supported by [95,98]. For this reason, a person-specific or episode-specific pre-FOG duration may help to reduce overlap with the walking class and increase class purity (contain only pre-FOG data), thus improving pre-FOG detection performance.

### 4.3. Features Used in FOG Detection and Prediction

A variety of features have been used in FOG detection and prediction. While most FOG detection and prediction features were previously established in non-PD applications [135,136,137,138], custom features were created to detect FOG, namely, freezing of gait criterion (FOGC) [46,47], freezing of gait detection on glasses (FOGDOG) [70], *k* value [59,60,61,62,72,73], *R* value [94], freeze index [29], *K* freeze index [67], and multichannel freeze index [67]. Time domain features, such as maximum acceleration amplitude within a window [40,41] or rotation about a single axis [98], are relatively simple and fast to compute. Gait-based features such as cadence [49], stride duration [35,50,71], and step length [35,71,77], as well as statistical features including mean [42,45,52,54,57,58,71,74,75,77,80,81,95], standard deviation [42,45,52,54,57,58,71,74,75,77,80,81,88,89,95,98], and root mean square [45,48,54,78,86] are also calculated from time domain data. Frequency domain features include freeze index (FI) [29], which was the most widely-used frequency domain feature [23,24,25,29,31,32,33,34,42,44,49,53,54,64,65,69,71,77,78,80,86,88,89,98], peak amplitude and corresponding frequency [40,41,64], standard deviation in frequency domain [50,57,64,74], spectral density centre of mass [50,57,66,74,80,81,86,96], and power of the signal in specific frequency bands [24,25,31,32,33,34,40,42,52,54,58,75,80]. While Fourier transforms are typically used to convert signals from the time domain to frequency domain, Fourier transform limitations have led to increased usage of wavelet approaches [51,56,63,71,79,83,91,92,93,96]. 

A feature set can be more representative of the wide range of FOG manifestations. Studies that combined time and frequency domains features [96] had better performance than either type of feature individually. Time domain features can account for gait parameters such as step length [35,71,77] cadence [49], asymmetry [45], and peak limb angular velocity [88,89], whereas frequency domain features can capture more subtle patterns characteristic of FOG, such as trembling in specific frequency bands [29]. The best performance is typically achieved with multiple features. 

The choice of features is very important, especially for real-time systems, where, in addition to classification performance considerations, classification speed is critical. For example, the calculation of stride duration at the end of the stride (approximately 1 s) could result in the delayed detection of a FOG event. Other features such as step length, cadence, cadence variation, stride peaks and FOGC may share this limitation, depending on the feature calculation method. Features extracted from appropriately-sized windowed data do not have this problem, since the features can be calculated as soon as the data window is available. The feature availability to the classifier is determined by the step size of the sliding window and calculation delay. All of the window-based features in Table 2 could be used in a real-time application, given sufficient processing power. However, an excessive number of features or complex features requiring many calculation stages may induce unacceptable delays when computing power is limited, as in many wearable systems. Using a minimal number of easily-calculated features is desirable; however, too few or overly-simple features may adversely impact classification performance. To address the delicate balance of classification performance versus classification speed, feature selection algorithms can be used to determine the best features from a larger set, as implemented in [45,51,55,56,58,66,67,76,80,83,86,95,96,98,99]. Algorithms such as the Relief-F or correlation-based approaches can be used to rank features according to their relevance so that the least relevant can be eliminated [139]. The most used feature selection methods in this review were paired *t*-tests [86,98], mutual information [45,58,67,95], and the Wilcoxon sum rank test [55,66,76,96]. The topic of feature selection is broad and encompasses numerous methods that can be used to improve classifier models. Given the diversity of features in the literature for detecting and predicting FOG, the best feature or feature set has yet to be determined. For future studies, it is generally suggested to begin with multiple features that can then be tuned or eliminated using feature section methods to produce a set of optimal features.

## 5. Conclusions

Based on 74 freezing of gait detection and prediction articles, this review reported details of the participants, walking task, sensors, features extracted, detection and prediction methods, and performance. The continued development of high-performing FOG detection methods is important for long-term monitoring and real-time cueing, and together with development of FOG prediction systems, is important for implementation in gait-assist systems. While FOG detection methods have been steadily increasing in performance, important challenges remain. Small FOG datasets may limit the machine-learning models that can be used, especially for deep learning. Sets of diverse features in both the time and frequency domains have helped to represent the inconsistent nature of FOG. The adoption of transfer learning, and semi-supervised learning models, built upon the established FOG detection methods, could add an element of personalization while preserving the robust generalization of person-independent models, thus making them promising approaches for future FOG detection and prediction research. 

## Figures and Tables

**Table 1:** Characteristics of FOG detection studies and FOG prediction studies, using wearable sensors.

Source	Studied Population	Walking Task Performed	Sensor Type and Location	FOG Detection Method	Features	Classifier Performance	Real Time
Moore 2008 [29]	11 FOG-PD (7 froze), ON and OFF, 46 episodes	Lab, straight walking, 180° turns, narrow doorways, obstacle avoidance.	IMU (1) left shank	Freeze index (FI) with person- specific thresholds. 6 s windows, detection based on FOG episode occurrences.	E	Detected 89.1% of episode occurrences, 10% false positives	No
Zabaleta 2008 [30]	4 FOG-PD, ON and OFF	Lab, sit to stand, 90° and 180° turns, figure-eight, doorway navigation, obstacle avoidance.	IMU (6) heels, shanks, thighs	Multivariate linear discriminant analysis, frequency-based features. Person-specific, detection based on classification of individual 3 s windows.	E	Area under ROC curve. Average of all participants: 0.937	No
Jovanov 2009 [23]	4 HC, 1 UFOG-PD	Lab, sit to stand and walking.	IMU (1) right knee	FI [29], 0.32 s windows (64 samples at 200 Hz).	E	-	Yes
Bachlin 2009–2010 [24,31,32,33] *	10 FOG-PD (8 froze), 237 episodes	Lab, straight walking, 180° turns, random instructions to start, stop, and turn 360° in both directions. Simulated ADL (walk to room, return with glass of water)	Acc (3) left shank, left thigh, lower back	FI [29] with additional energy threshold to reduce false positives due to standing. 4 s windows with 0.5 s shift each step. Detection performance based on classification of windows with a 2 s tolerance.	E	Person-independent threshold: Sensitivity: 73.1% Specificity: 81.6%	Yes
Bachlin 2009 [34] *	10 FOG-PD (8 froze) 237 episodes	Lab, straight walking, 180° turns, randomly given instructions and simulated ADL.	Acc (3) left shank, left thigh, lower back	Same methods as [24]. Improved offline through person-specific thresholds. Detection performance based on classification of windows with a 2 s tolerance.	E	Sensitivity: 88.6% Specificity: 92.8%	No
Delval 2010 [35]	10 HC, 10 NFOG-PD, 10 FOG-PD (5 froze), OFF, 20 episodes	Lab, 2 km/h treadmill, objects unexpectedly dropped on belt in front of participant.	CBMC, goniometers (2) knees	Compared stride features (e.g., step duration, step distance), and FI to person-independent thresholds, using 4.1 s windows.	E	Sensitivity: 75–83% Specificity: >95%	No
Djuric-Jovicic 2010 [36]	4 FOG-PD	Lab, sit to stand, straight walking through doorway, 180° turn, return to seat.	IMU (6) feet, shanks, thighs	Energy thresholds to detect movement, combined with NN for FOG detection. 0.2 s and 1.0 s windows. Classification performance based on number and duration of false detections.	E	Classification error up to 16%	No
Popovic 2010 [37]	9 FOG-PD (7 froze), ON, 24 episodes	Lab, sit to stand, straight walking through doorway, 180° turn, return to seat.	FSR in-shoe insole, Acc (6) feet, shanks, thighs	FSR signals to create single person-specific “normal step”. Pearson’s correlation coefficient (PCC) calculated for FSR signal of entire trial, then compared to a threshold.	E	-	No
Cole 2011 [38]	2 HC, 10 UFOG-PD, 107 episodes	Lab, unscripted ADL in mock apartment.	Acc (3) shin, thigh, forearm, EMG (1) shin	Stand vs sit detection, NN for FOG detection. Person-independent model, 2 s windows, detection performance calculated per 1 s segments.	E	Sensitivity: 82.9% Specificity: 97.3%	No
Tsipouras 2011 [39]	5 HC, 6 NFOG-PD, 5 FOG-PD	-	Acc (6) wrists, legs, chest, waist, Gyro (2) chest, waist	C4.5 decision tree, random forest, using 2 s windows.	E	Accuracy: Decision tree: 95.08% Random forest 96.11%	No
Niazmand 2011 [40]	6 FOG-PD (varying severity)	Lab, walk with 180° turns, with and without walking aid. Walking, 180° and 360° turns (both directions), doorways.	Instrumented pants, Acc (5) waist, thighs, shanks	Multi-stage, person-independent, threshold-based classification, identifies suspicious movement, then frequency feature for classification, using 2 s windows.	E	Sensitivity: 88.3% Specificity: 85.3%	No
Zhao 2012 [41]	8 FOG-PD (6 froze), 82 episodes	Lab, 5-8 min random instructions (stand, walk, stop, turn).	Instrumented pants, Acc (5) waist, thighs, shanks (as in [40])	Time series, acceleration peaks detection (1.5 s windows) and frequency features via FFT (4 s windows), compared to person-independent thresholds.	E	Sensitivity: 81.7%	No
Mazilu 2012 [42] *	10 FOG-PD (8 froze), 237 episodes	Lab, straight walking, 180° turns, randomly given instructions and simulated ADL.	Acc (3) left shank, left thigh, lower back	AdaBoosted decision tree classifier best among several. Compared window sizes 1–4 s, 1 s was ideal. Detection performance based on classification of individual windows.	E	Person-specific: Sensitivity: 98.35% Specificity: 99.72% Person-independent: Sensitivity: 66.25% Specificity: 95.38%	No
Tripoliti 2013 [43]	5 HC, 6 NFOG-PD, 5 FOG-PD, ON and OFF, 93 episodes	Lab, rise from bed, walking tasks including doorways, 180° turns, and ADL.	Acc (4) ankles, wrists, IMU (2) waist, chest	Random forest classifier, 1 s windows. Person-independent detection performance based on classification of individual windows.	E	Sensitivity: 81.94% Specificity: 98.74%	No
Moore 2013 [44]	25 FOG-PD (20 froze), OFF, 298 episodes	Lab, TUG.	IMU (7) Lower back, thighs, shanks, feet	FI thresholds [29]. Compared different sensor locations, person-independent thresholds and window lengths. Detection performance based on classification of FOG episode occurrences and percentage of time frozen.	E	Lower back sensor, 10 s window: Sensitivity: 86.2% Specificity: 82.4%	No
Mazilu 2013 [45] *	10 FOG-PD (8 froze), 237 episodes	Lab, straight walking, 180° turns, random instructions and simulated ADL.	Acc (3) left shank, left thigh, lower back	Person-specific decision tree, tested different feature sets and supervised vs unsupervised feature selection using principal component analysis (PCA). Detection performance based on classification of individual 1 s windows.	E, S	Unsupervised: Sensitivity: 77.7% Specificity: 87.56% Supervised: Sensitivity: 69.42% Specificity: 87.76%	No
Coste 2014 [46]	4 UFOG-PD, 44 episodes	Lab, corridor walk with dual task.	IMU (1) shank	Freezing of gait criterion (FOGC) feature, based on cadence and stride length, incorporating person-specific thresholds. Detection performance based on classification of FOG episode occurrences.	E	Sensitivity: 79.5%	No
Sijobert 2014 [47]	7 UFOG-PD, 50 episodes	Lab, corridor walk with dual task.	IMU (1) shank	FOGC [46], with person-specific thresholds. Detection performance based on classifying FOG episode occurrences. FOG episodes labeled as Green (n = 19, slight gait modification with no fall risk), Orange (n = 12, gait modification with fall risk) or red (n = 19, FOG – blocked gait).	E	Correctly identified 26 of 31 FOG (orange and red)	No
Kwon 2014 [48]	20 FOG-PD (6 froze), ON, 36 episodes	Lab, repeated straight walk with 180° turns.	Acc (1) in shoe heel	Root mean square (RMS) of acceleration compared to person-specific threshold. 0.2–10 s windows. 3–4 s windows recommended.	E	Minimum of sensitivity or specificity: 85.8%	No
Pepa 2014 [49]	18 UFOG-PD, ON	Lab, 3 TUG variations: standard, with cognitive dual task, with manual dual task.	Acc (1) smartphone worn on belt at hip	Fuzzy logic model using frequency features, person-specific thresholds, 2.56 s windows. Detection performance based on classification of windows (sensitivity, specificity) and FOG episode occurrences (sensitivity) – distinction not indicated in results.	E	Sensitivity: 89% Specificity: 97%	No
Djuric-Jovicic 2014 [50]	12 FOG-PD, OFF	Lab, sit to stand, walk with 90° and 180° turns, multiple doorways.	IMU (2) shanks, FSR in-shoe insoles	Each stride is compared to a “normal” stride using spectral power, stride duration, and shank displacement. Custom rule-based method classified each stride based on person-specific thresholds.	E	FOG with tremor: Sensitivity: 99% Specificity: 100% FOG complete stop: Sensitivity: 100% Specificity: 100%	No
Assam 2014 [51] *	10 FOG-PD (8 froze), 237 episodes	Lab, straight walking, 180° turns, random instructions and simulated ADL.	Acc (3) left shank, left thigh, lower back	Wavelet decomposition for feature extraction and conditional random fields for classification. Train/test for each person individually (person-specific model), compared 2.5, 4 and 8 s windows. Results for 3 participants, separately.	E, S	Best single participant results, with 4s window: Sensitivity: 65% Precision: 61.9%	No
Mazilu 2014 [25]	5 FOG-PD, 102 episodes	Lab, walking with turns and doorways.	IMU (2) ankles	Person-independent decision tree classifier (C4.5), multiple frequency-based input features, 2 s windows. Detection performance based on classifying FOG episode occurrences.	E	99 of 102 FOG detected	Yes
Mazilu 2015 [52] **	18 FOG-PD (11 froze), 182 episodes	Lab, walking tasks with cognitive and manual tasks. Straight walking, 180° and 360° turns, narrow spaces, hospital circuit with elevator, unexpected stops start, and turns.	IMU (2) wrists	Decision tree classifier (C4.5), features from wrist data, 3 s windows, person-specific detection performance based on classifying FOG episode occurrences.	E	Person-specific: Sensitivity: 90% Specificity: 83%	No
Zach 2015 [53]	23 FOG-PD (16 froze), OFF, 166 episodes	Lab, self-paced, fast walking, short steps, short fast steps, 360° turns both directions.	Acc (1) lower back	FI [29] compared to person-specific and person-independent thresholds, 2 s windows, detection performance based on classifying FOG episode occurrences.	E	Person-independent threshold: Sensitivity: 75% Specificity: 76%	No
Kim 2015 [54]	15 FOG-PD (9 froze), 46 episodes	Lab, hospital hallway, straight walk with 180° turns, also with dual tasks.	IMU (1) (smartphone) ankle, pants pocket, chest pocket, waist	Adaboosted, person-independent, decision tree using 4 s windows. Compared different sensor locations, found waist best.	E	Smartphone on waist: Sensitivity: 86% Specificity: 91.7%	No
Handojoseno 2015 [55]	4 FOG-PD, OFF	Lab, TUG with 180° or 540° turns in both directions.	EEG, head	Person-independent NN to detect FOG during turning, 0.256 s windows, 1 s samples (117 normal turning, 224 FOG turning).	E, S	Sensitivity: 74.6% Specificity: 48.4%	No
Venu 2016 [56] *	10 FOG-PD (8 froze), 237 episodes	Lab, straight walking, 180° turns, random instructions and simulated ADL.	Acc (3) left shank, left thigh, lower back	Wavelet decomposition used sub-band energies as features, continuous random field used for detection. 4 s windows. Person-independent detection performance based on classifying FOG episode occurrences.	E, S	Average of 3 participants test set: Sensitivity: 90.3% Precision: 95.8%	No
Martin 2016 [57] ****	6 FOG-PD, ON and OFF	Participant’s home, 180° turns, doorways, walking outside, dual tasking and false positive test intended to create shaking resembling FOG (e.g., brushing teeth).	Acc (1) left hip	Different methods, feature sets, and window sizes compared. Best results from SVM. Detection performance based on classification of individual 1.6 s windows.	E	Sensitivity: 91.7% Specificity: 87.4%	No
Mazilu 2016 [58] **	18 FOG-PD (11 froze), 184 episodes	Lab, walking tasks with cognitive and manual tasks. Straight walking, 180° and 360° turns, narrow spaces and hospital circuit with elevator, unexpected stops start, and turns.	IMU (2) wrists	Decision tree classifier (C4.5) similar to [52], but fewer features and evaluation of single wrist input. 3 s windows, detection performance based on classifying FOG episode occurrences.	E, S	Person-specific: Sensitivity: 85% Specificity: 80% Person-independent: Sensitivity: 90% Specificity: 66%	No
Lorenzi 2016 [59,60,61,62]	16 UFOG-PD	Lab, walking through doorway, 180° turns.	IMU (2) shanks, IMU (1) side of head	Compared headset (combined with NN) and shin mounted IMUs. Shin method using custom *k*-index feature compared to person specific thresholds performed best.	E	From shin system: Sensitivity: 94.5% Specificity: 96.7%	No
Rezvanian 2016 [63] *	10 FOG-PD (8 froze), 237 episodes	Lab, straight walking, 180° turns, random instructions and simulated ADL.	Acc (3) left shank, left thigh, lower back	Continuous wavelet transform computed ratio of frequency ranges, compared to person-independent threshold. Compared different window lengths, suggested 2 s windows for future real-time implementation.	E	Window 2 s: Sensitivity: 82.1% Specificity: 77.1% Window 4 s: Sensitivity: 84.9% Specificity: 81.01%	No
Ahlrichs 2016 [64] ***	20 FOG-PD (8 froze) ON and OFF, 209 episodes	Participant’s home, 180° turns, doorways, walking outside, dual tasking and a false positive test intended to create shaking resembling FOG (e.g., brushing teeth).	Acc (1) waist	Person-independent SVM (linear kernel), best results with 3.2 s windows. Classified windows aggregated over 60 s and degree of confidence calculated and compared to threshold to determine whether a FOG episode was present during aggregation period.	E	Sensitivity: 92.3% Specificity: 100%	No
Capecci 2016 [65]	20 FOG-PD (16 froze), ON, 98 episodes	Lab, TUG test, cognitive or manual dual task.	IMU (1) smartphone at waist	Cadence and modified freeze index extracted and compared to person-specific thresholds. Detection performance based on classification of individual 3.56 s windows.	E	Sensitivity: 87.57% Specificity: 94.97%	No
Ly 2016 [66]	7 FOG-PD, OFF	Lab, TUG.	EEG, head	Person-independent NN, compared different features and number of EEG channel inputs. Data divided into 1 s segments (343 effective walking and 343 freezing).	E, S	Using all 32 channels: Sensitivity: 72.2% Accuracy: 71.46%	No
Pham 2017 [20] *	10 FOG-PD (8 froze), 237 episodes	Lab, straight walking, 180° turns, random instructions and simulated ADL.	Acc (3) left shank, left thigh, lower back	Anomaly detection approach. Acceleration and spectral coherence features calculated for incoming window and “normal” reference. Person-independent thresholds used to classify FOG, “normal” reference updated with each non-FOG window. Detection performance based on classification of individual 0.6 s windows.	E	Sensitivity: 87% Specificity: 94%	No
Pham 2017 [67] *	Development: 10 FOG-PD (8 froze), Test: 24 FOG-PD (OFF)	Lab, straight walking, 180° turns, random instructions and simulated ADL. Test: TUG, 180° and 540° turns in both directions.	Acc (3) left shank, left thigh, lower back IMU (7) foot, shank, thigh, lower back/hip	Development data from Daphnet*, test data from [68]. Several new features (including multichannel freeze index) presented and evaluated, detection used anomaly score compared to person-independent threshold to classify individual 3 s windows.	E, S	Freeze index using hip sensor X-axis: Sensitivity: 89% Specificity: 94%	No
Pham 2017 [69] *	10 FOG-PD (8 froze), 237 episodes	Lab, straight walking, 180° turns, random instructions and simulated ADL.	Acc (3) left shank, left thigh, lower back	Freezing index and spectral coherence features used to generate average value used as threshold for FOG detection. Participant independent averages automatically updated during use. Detection performance based on classification of 0.6 s windows.	E	Sensitivity: 89.2% Specificity: 95.6%	No
Ahn 2017 [70]	10 HC, 10 FOG-PD, OFF, 42 episodes	Lab, TUG and 10 m walk tests.	IMU (1) in smart glasses	Custom FOG detection on glasses feature (FOGDOG), incorporated stride length and cadence, with person-specific thresholds, 1 s windows. Detection performance based on classifying FOG episode occurrences	E	For PD participants: Sensitivity: 97% Specificity: 88%	Yes
Tahafchi 2017 [71]	2 FOG-PD	Lab, 6 min of walking turning and stepping in place.	EMG + IMU units (6) thighs, shanks, feet	SVM with Gaussian kernel, multiple time series and frequency features. 1 s windows.	E	Sensitivity: 90% Specificity: 92%	No
Suppa 2017 [72]	28 FOG-PD (25 froze), 152 episodes (102 OFF, 50 ON)	Lab, simulated home environment, TUG passing into narrow hall, turning both directions.	IMU (2) shins	*k* index from shin-mounted sensor compared to person-specific thresholds [59], with additional analysis of ON vs. OFF states.	E	Sensitivity: 93.41% Specificity: 98.51%	No
Kita 2017 [73]	32 UFOG-PD (25 froze)	Lab, straight walking, through doorway, with 180° turn, and return.	IMU (2) shanks	Improvements on *k* index in [59], including new *K_swing_*, *K’* features. Person-specific performance based on percentage of time frozen per trial.	E	Sensitivity: 93.41% Specificity: 97.57%	No
Rodriguez-Martin 2017 [74] ***, ****	21 FOG-PD, ON and OFF, 1321 episodes	Participant’s home, 180° turns, doorways, walking outside, dual tasking and a false positive test intended to create shaking resembling FOG (e.g., brushing teeth).	IMU (1) left hip	SVM (radial basis function kernel), compared person-independent and person-specific models, using 3.2 s windows. Detection performance based on classifying FOG episode occurrences.	E	Person-independent: Sensitivity: 74.7% Specificity: 79.0% Person-specific: Sensitivity: 88.09% Specificity: 80.09%	No
Rodriguez-Martin 2017 [75] ***, ****	12 PD-FOG, 106 episodes	Participant’s home, 180° turns, doorways, walking outside, dual tasking and a false positive test intended to create shaking resembling FOG (e.g., brushing teeth).	IMU (1) left hip	Same detection algorithm as [74], also using 3.2 s windows. Detection performance based on classifying FOG episode occurrences.	E	Sensitivity: 82.08% Specificity: 97.19%	Yes
Ly 2017 [76]	6 FOG-PD	Lab, TUG.	EEG, head	Person-independent Bayesian NN, to detect FOG during turns. Similar to [55], with addition of S-transform. Data divided into 1 s samples (204 normal turning, 204 FOG turning).	E, S	Sensitivity: 84.2% Specificity: 88.0%	No
Pepa 2017 [77]	20 UFOG-PD	Lab, TUG, with cognitive or manual dual task, sit, lay on bed, stand up and maintain upright posture, and run on a treadmill if able.	IMU (1) smartphone at waist	Fuzzy inference system compared to person-specific thresholds to detect periods of walking and FOG. 2.56 s windows (256 samples at 100 Hz). Detection performance based on classifying FOG episode occurrences, duration of FOG also examined.	E	FOG detection performance using ANOVA.	Yes
Wang 2017 [78]	9 UFOG-PD, OFF	Lab, gait initialization, narrow aisle, turning and dual tasks. One participant performed ADL in their home.	Acc (1) lower back	FI and RMS of acceleration. Both compared to person-specific thresholds and combined with an OR statement. Detection performance calculated as percent time frozen per trial.	E	Sensitivity: 90.8% Specificity: 91.4%	No
Punin 2017 [79]	1 HC, 1 NFOG-PD, 6 FOG-PD, OFF, 27 episodes	Lab, stair climb and descent, straight walking and 180° turns.	IMU (1) right ankle	Discrete wavelet transform, compared to person-independent threshold. Detection performance based on classifying FOG episode occurrences.	E	Sensitivity: 86.66% Specificity: 60.61%	Yes
Saad 2017 [80]	5 FOG-PD ON, 64 episodes	Lab, straight walking, 180° turn, manual dual task or narrowed walking path. Clinic circuit including unscripted stops, starts, turns and doorways.	Acc (2) foot, shin, Goniometer (1) knee, Telemeters (IR proximity sensors) (2) upper and lower medial shank	Time and frequency domain features extracted from 2 s windows. Best features for each sensor identified. Person-independent, NN with Gaussian activation function used for detection. Defined average performance as mean of the fraction of FOG correctly identified and the fraction of non-FOG correctly identified.	E, S	Average of all participants: Performance: 87%	No
Sama 2018 [81] ****	15 FOG-PD, ON and OFF	Participant’s home, 180° turns, doorways, walking outside, dual tasking and a false positive test intended to create shaking resembling FOG (e.g. brushing teeth).	IMU (1) left hip	Compared multiple classifiers and feature sets, best results with SVM, using 1.6 s windows (64 samples at 40 Hz). Person-independent detection performance based on classifying FOG episode occurrences	E	Sensitivity: 91.81% Specificity: 87.45%	No
Prateek 2018 [82]	16 UFOG-PD (8 froze), 58 episodes	Lab, walking backwards, 180° turns, stepping over a board, walk a figure-eight loop, walk between sets of chairs placed close together.	IMU (2) heels	Detect instances of zero velocity or trembling, then, a point process filter computed probability of FOG based on foot position, orientation, and velocity. Detection performance based on classifying FOG episode occurrences, duration of FOG also examined.	E	Person-specific model, detected 47/58 FOG episode occurrences. Accuracy: 81.03%	No,
Ashour 2018 [83] *	4 participants from Daphnet	Lab, straight walking, 180° turns, random instructions and simulated ADL.	Acc (3) left shank, left thigh, lower back	SVM (linear kernel). Used infinite feature ranking [84] to reduce feature set. Person-specific detection performance based on classifying FOG episode occurrences.	E, S	1 patient top ranked (30 features) Accuracy: 94.4%	No
Camps 2018 [85] ****	21 FOG-PD, ON and OFF	Participant’s home, 180° turns, doorways, walking outside, dual tasking and a false positive test intended to create shaking resembling FOG (e.g., brushing teeth).	IMU (1) left hip	1D CNN, 2.56 s windows stacked to combine current and previous windows. Person-independent detection performance based on classification of windows. Replicated other FOG detection methods and compared performance of models and feature sets.	-	CNN: Sensitivity: 91.9% Specificity: 89.5%	No
Oung 2018 [86] *	10 FOG-PD (8 froze), 237 episodes	Lab, straight walking, 180° turns, random instructions and simulated ADL.	Acc (3) left shank, left thigh, lower back	Probabilistic NN, using time domain features (117) and frequency features (126), 4 s windows. Also examined SVM with RBF kernel. Person-specific and person-independent models compared.	E, S	Person-specific: Sensitivity: 99.83% Specificity: 99.96% Person-independent: Sensitivity: 87.71% Specificity: 87.38%	No
Li 2018 [87]	10 FOG PD, OFF, 281 episodes	Lab, straight walking (10 m and 100 m), 180° turns, narrow spaces.	Acc (1) lower back	Person-independent, unsupervised approach (training data not labelled). Mini batch *k* means clustering algorithm using acceleration entropy, 1 s windows. Once the centre of the FOG and non-FOG classes were found, new data were classified based on which centre was closest.	E	Sensitivity: 92.4% Specificity: 94.9%	No
Mikos 2018 [88,89]	25 people, no other description provided (23 froze), 221 episodes	Lab, TUG and random walking.	IMU (2) ankles	Semi-supervised approach. NN, base training person-independent. Then unsupervised training during use improved performance.	E	Sensitivity: 95.9% Specificity: 93.1%	Yes
Rad 2018 [90] *	10 FOG-PD (8 froze), 237 episodes	Lab, straight walking, 180° turns, random instructions and simulated ADL.	Acc (3) left shank, left thigh, lower back	Probabilistic anomaly detection approach using denoising autoencoder. Person-independent model trained to recognize normal gait (trained using non-FOG data), 1 s windows. Compared CNN trained using non-FOG (unsupervised) and FOG (supervised) data for comparison.	-	Proposed model: AUC: 77% Supervised model: AUC: 84%	No
El-Attar 2019 [91] *	10 FOG-PD (8 froze), 237 episodes	Lab, straight walking, 180° turns, random instructions and simulated ADL.	Acc (1) left shank	Combined 1D discrete wavelet transform with FFT features, and used NN for classification. Person-specific detection performance based on classifying FOG episode occurrences.	E	Accuracy: 96.3%	No
Punin 2019 [92,93]	1 HC, 1 NFOG-PD, 6 FOG-PD, 27 episodes	Lab, straight walking, 180° turns, stair climbing.	IMU (2) back of ankles (distal posterior shank)	Discrete wavelet transform, signal energy compared to person-independent threshold using 32 s windows (256 samples at 8 Hz), updated every second. Detection performance based on classifying FOG episode occurrences.	E	Sensitivity: 60.61% Specificity: 86.66%	Yes
Mazzetta 2019 [94]	7 PD with varying disease severity, tested ON and OFF	Simulated apartment, TUG turning both ways, narrow hallways and doorways.	IMU/EMG devices shanks (tibialis anterior, gastrocnemius medialis)	Multi-stage thresholds using gyroscope and surface EMG. Gyro signal and threshold used to identify beginning and end of each step, then custom *R* feature compared to person-independent threshold distinguished FOG. Detection performance based on classifying individual steps.	E	False positive rate 5% False negative rate 2%	No
FOG Prediction							
Mazilu 2013 [45] *	10 FOG-PD (8 froze), 237 episodes	Lab, straight walking, 180° turns, random instructions and simulated ADL.	Acc (3) left shank, left thigh, lower back	Assumed duration of pre-FOG class (1–6 s). 3 class decision tree classifier (pre-FOG, FOG, not FOG) and 1 s window for feature extraction. Person-specific, prediction performance based on classification of individual windows.	E, S	1 participant with assumed 3 s pre-FOG F1-score: 0.56	No
Mazilu 2015 [95] **	11 FOG-PD	Lab, walking with cognitive and manual tasks: straight, 180° and 360° turns, narrow spaces and hospital circuit involving elevator, unexpected stops start and turns.	Electrocardio-gram (1) (ECG) chest, galvanic skin response (1) (fingertip)	Assumed Pre-FOG duration (3 s) used for feature selection. Feature extraction used 3 s window. Multivariate Gaussian distribution used in anomaly detection model. Person-specific model for each individual. Instead of pre-defined pre-FOG length, model decision threshold set manually. Prediction based on number of FOG episode occurrences.	E, S	SC data predicted 132/184 (71.3%) of FOG episode occurrences on average 4.2 s in advance, 71 false positives.	No
Handojoseno 2015 [96]	16 FOG-PD, 404 episodes	Lab, TUG.	EEG, head	Person-independent NN trained with 462, 1 s data segments for each class, tested on 172 segments. Extracted multiple frequency-based features using FFT and wavelets, multilayer perceptron NN for classification. Defined pre-FOG as data between 5 s and 1 s prior to FOG.	E, S	Sensitivity: 86% Precision: 74.4%	No
Zia 2016 [97] *	3 chosen randomly from Daphnet	Lab, straight walking, 180° turns, random instructions and simulated ADL.	Acc (1) left shank	Person-specific layered recurrent NN. Detection applied to the 5 s prior to FOG. One participant had best results, trained on 9 episode occurrences, tested on 15.	-	Best participant: Sensitivity: 30% Precision: 89%	No
Palmerini 2017 [98] **	18 FOG-PD (11 froze), 180 episodes	Lab, walking with cognitive and manual tasks: straight, 180° and 360° turns, narrow spaces and hospital circuit involving elevator, unexpected stops start and turns.	IMU (3) ankles, lower back	Assumed pre-FOG as 2 s before FOG. Features extracted from 2 s windows. Linear discriminant analysis to classify pre-FOG vs normal gait windows. Person-independent model.	E, S	Sensitivity: 83% Specificity: 67%	No
Handojoseno 2018 [99]	16 FOG-PD	Lab, TUG.	EEG, head	Person-independent NN trained with 462, 1 s data segments for each class, tested on 172. Predict FOG by classifying data segment 5 s prior to freeze with Bayesian NN.	E, S	Sensitivity: 85.86% Specificity: 80.25%	No
Torvi 2019 [100] *	10 FOG-PD (8 froze), 237 episodes	Lab, straight walking, 180° turns, random instructions and simulated ADL.	Acc (3) left shank, left thigh, lower back	LSTM and RNN with 2 transfer learning approaches. Found best performance with LSTM, trained network then added person-specific final layer. Examined set pre-FOG duration: 1, 3 and 5 s.	-	Predicted FOG up to 5 s in advance with >90% accuracy	No

* Daphnet dataset originally collected by Bachlin et al. [24] (n = 10, 8 froze during testing). A total of 237 FOG episodes (8 participants OFF, 2 ON who claimed to freeze often while ON). Accelerometers on left shank, left thigh, and lower back. ** CuPiD dataset originally collected by Mazilu et al. [101] (n = 18, 11 froze during testing). 180 FOG episodes (ON/OFF state not mentioned in original article, subsequently reported ON state [58]). [52] reported 182 FOG episodes and [58] reported 184 episodes. IMU (9) on wrists, thighs, ankles, feet, and lower back. Galvanic skin response sensor (1) on hand, ECG sensor (1) on chest, smartphone (1) in front pocket with integrated IMU, pressure sensing shoe insole (1), functional near-infra-red spectroscopy (fNIR) sensor on forehead. *** REMPARK project (Personal Health Device for the Remote and Autonomous Management of Parkinson’s Disease) [102,103]. Data collected by multiple researchers, in participant’s homes in OFF and ON states. Waist worn IMU. **** MASPARK project [104]. Abbreviations and acronyms: Feature extraction (E), selection (S). FOG: freezing of gait; HC: healthy control participants; FOG-PD: people with PD with FOG symptoms; NFOG-PD: people with PD with no FOG symptoms; UFOG-PD: FOG symptoms not reported; ON: on medication, OFF: off medication; Acc: accelerometer, EEG: electroencephalogram; EMG: electromyography; Gyro: gyroscope; IMU: inertial measurement unit, CBMC: camera-based motion capture; CNN: convolutional neural network; NN: neural network; RNN: recurrent neural network; LSTM: long short-term memory neural network; SVM: support vector machine; ADL: activities of daily living; TUG: Timed Up and Go Test; AUC: area under ROC curve; FFT: fast Fourier transform; FI: freeze index [29]; FOGC: freezing of gait criterion; FSR: force sensing resistor; GSR/SC: galvanic skin response/skin conductance; PCA: principal component analysis; PCC: Pearson correlation coefficient; PSD: power spectral density; RMS: Root mean square; ROC: receiver operating characteristic.

**Table 2:** Features extracted from wearable-sensor data and used for freezing of gait detection or prediction.

Feature Name	Sensor Type	Sensor Location	Feature Description	Source
Mean	Acc, Gyro GSR Goniometer Telemeters	Chest, wrist, lower back, waist, thigh, knee, shanks, ankle, foot, GSR: finger, Goniometers: knees, Telemeters: between shanks	Mean of signal within window and axis. Acceleration: 3D vector magnitude or 3 axes Gyro: Angular velocity 3D vector magnitude, or 3 axes GSR: Conductance, low-pass filtered at 0.9 Hz Goniometer: Knee angular rotation. Telemeter: Voltage output, spikes in signal indicate that legs are next to one another.	[42,45,52,54,57,58,71,74,75,77,80,81,95]
Min, Max, Median, HarmMean, GeoMean, Trim mean, Mode, Range	Acc, GSR	Shank, thigh, lower backGSR: finger	Descriptive statistics within given window. Acceleration: 3D vector magnitude, or individual axes GSR: Conductance, low-pass filtered at 0.9 Hz	[45,64,75,95]
Increment of mean values	Acc	Waist	Difference between mean of current window and mean of previous window for anterior/posterior acceleration.	[57,74,81]
Difference in means of different axes	Acc	Waist	Difference in acceleration mean values between axes for current window (X and Y, X and Z, Y and Z).	[57,81]
Number of peaks in a window	Acc	Instrumented pants, Acc (5) waist, thighs and shanks	Number of times relative acceleration signal [105] passes above a threshold during 1.5 s window. Normal reference set to 3. More than 3 peaks per 1.5 s considered possible FOG.	[40,41]
Duration of acceleration above threshold	Acc	Instrumented pants, Acc (5) waist, thighs and shanks	Time the relative acceleration signal [105] is above a threshold. Normal reference 0.85 s per 1.5 s window. Longer durations considered suspicious (possibly FOG).	[40,41]
Turning degrees	Gyro	Lower back	Angular rotation about vertical axis. Calculated as the integral of low pass filtered (1.5 Hz) angular velocity about the vertical axis.	[98]
Left-right cross-correlation	Gyro	Ankles	Maximum cross-correlation between mediolateral angular velocity (de-trended), left and right ankles (0.25 to 1.25 s).	[98]
Left-Right average SD	Gyro	Ankles	Average between SD of mediolateral angular velocity (de-trended), of right and left ankles.	[98]
RMS	Acc, Gyro	Sole of shoe, shank, thigh, low back, ankle, chest	Root mean square (RMS) of acceleration or angular velocity data in given window, for 3 axes.	[45,48,54,78,86]
Inter quantile range	Acc, Gyro	Ankle, thigh, chest, and waist	Interquartile range of acceleration or angular velocity in given window, for 3 axes.	[54]
Standard deviation	Acc, Gyro GSR Goniometer (G) Telemeters (T)	Chest, lower back, waist, thigh, shanks, ankle, foot, wrist, GSR: finger G: kneesT: between shanks	Standard deviation in given window. Acceleration: 3D vector magnitude or 3 axes Gyro: 3D vector magnitude of angular velocity, or 3 axes GSR: Conductance, low-pass filtered at 0.9 Hz Goniometer: Knee angular rotation. Telemeter: Voltage output, spikes in signal indicate that the legs are next to one another.	[42,45,52,54,57,58,71,74,75,77,80,81,88,89,95,98]
Variance	Acc, Gyro	Shanks, thigh, lower back, waist, ankle, chest	Variance in given window. Calculated for acceleration or angular velocity data in given window, for 3 axes. In [83] and [91], variance calculated for FFT signal and detail and approximation coefficients from discrete wavelet transform.	[42,45,54,83,91]
Acceleration indicator ($S_{AC}$)	Acc	Shank, thigh, lower back	Binary value, to detect acceleration in each axis $S_{AC}=sgn(\left(X-\left(\bar{X}-\sigma \right){)}_{+}\right)$, where *X* is a set of acceleration data, $\bar{X}$ is mean of X, σ is standard deviation of *X*, and *sgn(a)* is a sign function of *a* while *(a)_+_* returns *a* only if *a* ≥ 0, otherwise returns 0.	[20]
Zero velocity and Trembling event intervals (ZVEI, TREI)	Acc, Gyro	Heel	Direction of gravitational acceleration used to calculate ZVEI and TREI to determine if foot is stationary (zero velocity) or trembling, from all acceleration and angular velocity axes.	[82]
Foot speed	Acc, Gyro	Heel	Foot position, orientation, and velocity, from 3 axis acceleration and angular velocity [106].	[82]
Integral	Acc	Waist, shank, thigh, low back	Integral of acceleration in given window, for given axis.	[57,74,81,86]
Kurtosis	Acc, Gyro	Waist, ankle, shank, thigh low back	Kurtosis within a given window, from all acceleration axes, angular velocity, acceleration 3D vector, or absolute value of harmonics in 0.04–0.68, 0.68–3 and 3–8 Hz frequency bands (calculated from FFT of 3D acceleration)	[45,54,57,74,75,81]
Skewness	Acc	Waist, shank, thigh, low back	Measure of signal asymmetry within a given window, from all axes of the acceleration, angular velocity, acceleration 3D vector magnitude, or absolute value of harmonics in 0.04–0.68, 0.68–3 and 3–8 Hz frequency bands (calculated from FFT of 3D acceleration).	[45,57,74,75,81]
Mean absolute Value	Acc	Shank, thigh, low back	$MAV=\frac{1}{N}\sum_{n=1}^{N}\left|x_{n}\right|$ For acceleration *x* within a window of *N* data points. Calculated for 3 axes.	[86]
Simple square interval	Acc	Shank, thigh, low back	$SSI=\sum_{n=1}^{N}{\left|x_{n}\right|}^{2}$ For acceleration *x* within a window of *N* data points. Calculated for 3 axes.	[86]
v-order 2 and 3	Acc	Shank, thigh, low back	$v2={\left(\frac{1}{N}\overset{N}{\underset{i=1}{\sum }}x_{i}^{2}\right)}^{\frac{1}{2}},\;v3={\left(\frac{1}{N}\sum_{i=1}^{N}{\left|x\right|}_{i}^{3}\right)}^{\frac{1}{3}}$ For acceleration *x* within window of *N* data points. Calculated for 3 axes.	[86]
Waveform length	Acc	Shank, thigh, low back	$WL=\sum_{n=1}^{N-1}\left|x_{n+1}-x_{n}\right|$ For acceleration *x* within window of *N* data points. Calculated for 3 axes.	[86]
Average amplitude change	Acc	Shank, thigh, low back	$AAC=\frac{1}{N}\sum_{n=1}^{N-1}\left|x_{n+1}-x_{n}\right|$ For acceleration *x* within a window of *N* data points. Calculated for 3 axes.	[86]
Difference absolute standard deviation	Acc	Shank, thigh, low back	$DASDV=\sqrt{\frac{1}{N-1}\sum_{n=1}^{N-1}{\left(x_{n+1}-x_{n}\right)}^{2}}$ For acceleration *x* within window of *N* data points. Calculated for 3 axes.	[86]
Maximum fractal length	Acc	Shank, thigh, low back	$MFL=log_{10}\left(\sqrt{\sum_{n=1}^{N-1}{\left(x_{n}-x_{n+1}\right)}^{2}}\right)$ For acceleration *x* within window of *N* data points. Calculated for 3 axes.	[86]
Step length	Acc, CBMC	Waist, thigh, shank, foot	Distance (m) between consecutive footfalls of the same limb, measured as double integral of A/P acceleration or by camera-based motion capture.	[35,71,77]
Step duration	Gyro	Thigh, shank, ankle, foot	Duration (s) between consecutive footfalls of same limb, calculated from angular velocity peaks (raw or filtered)	[35,50,71]
Cadence	Acc, Gyro	Feet, shank, thigh, waist	Number of steps in given time (e.g., steps/minute), from time between peaks in angular velocity, vertical acceleration, second harmonic of acceleration in frequency domain [65], or calculated as in [107].	[35,49,65,77]
Cadence variation	Acc	Waist	Standard deviation of cadence, from last 3 windows.	[49]
Stride peaks	Gyro, Angular velocity	Shank (ankle)	Peak of low pass filtered (4^th^ order Butterworth 10 Hz) angular velocity within gait cycle, in frontal plane.	[88,89]
Zero Crossing rate, mean crossing rate	Acc	Shank, thigh, low back	Number of times acceleration signal changes between positive and negative. Number of times acceleration signal changes between below average and above average in a given window. Calculated for 3 axes.	[45]
Signal vector magnitude	Acc	Shank, thigh, low back	Summation of Euclidean norm over 3 axes over entire window, normalized by window length.	[45]
PCA	Acc Goniometer (G) Telemeters (T)	Waist, shank, thigh, low back G: knees T: between shanks	Principal component analysis, calculated from raw 3 axis acceleration data from all sensors, each acceleration axis within specific spectral bands, or used to decrease dimensionality of multi-sensor feature set.	[45,74,80,81]
Normalized signal magnitude area (SMA)	Acc	Shank, thigh, low back	Acceleration magnitude summed over 3 axes normalized by window length.	[45,75]
Eigenvalues of dominant directions (EVA)	Acc	Shank, thigh, low back	Eigenvalues of covariance matrix of acceleration along all 3 axes.	[45]
Energy (time domain)	Acc, Gyro, EMG on tibialis anterior	Forearm, foot, shank and thigh, waist, EMG: on shin	Energy, where *x(n*) is discrete signal in time domain, *n* sample index, *T* window length, and *E* signal energy: $E=\sum_{n=1}^{T}{\left|x\left(n\right)\right|}^{2}$ Calculated from each acceleration or angular velocity axis, or from surface EMG signal.	[36,38]
Average acceleration energy (AAE)	Acc	Shank, thigh, low back	Mean of acceleration signal energy over 3 axes.	[45]
Asymmetry coefficient	Acc	Shank, thigh, low back	The first moment of acceleration data in window divided by standard deviation over window. Calculated for 3 axes.	[45]
Freezing of gait criterion (FOGC)	Gyro, Acc	Shank	Cadence and stride length measure, for stride *n* $$FOGC_{n}=\frac{C_{n}\;L_{min}}{C_{max}\left(L_{n}+L_{min}\right)}$$ where $C_{n}$ is cadence, $L_{n}$ stride length. Maximum cadence $C_{max}$ set to 5 strides/s, and minimum stride length $L_{min}$ = 5 cm. Cadence and stride parameters calculated from angular velocity and acceleration [108]	[46,47]
FOG detection on glasses (FOGDOG)	Acc	Head	$$FOGDOG=\frac{N_{step}}{N_{max}}\;\frac{\left(D_{ref}-D^{\prime }\right)}{D_{ref}}$$ where $D^{\prime }$ is cumulative forward distance travelled by person during window, $D_{ref}$ pre-set normal forward distance travelled, $N_{step}$ cadence (number of steps/s), $N_{max}$ pre-set maximum normal cadence, forward distance from double integral of forward acceleration after correction for head tilt angle, step length from [109].	[70]
K index, and K’ index	Gyro	Shank	Summation of absolute value of low pass filtered angular velocity of left and right shanks in sagittal plane: $$k\;=lowpass\left(\left|\omega_{left}\right|\right)\;+lowpass\left(\left|\omega_{right}\right|\right)$$ $\omega_{left}$ and $\omega_{right}$ are angular velocities in sagittal plane.	[59,60,61,62,72,73]
*R* value	Gyro, angular velocity	IMU/EMG devices on shanks (tibialis anterior and gastrocnemius medialis)	*R* value is calculated once for each stride. $$R=\frac{\max\left(ABS\right)}{{sEMG|}_{t=t_{\max\left(ABS\right)}}}$$ ABS is absolute value of moving average angular velocity in sagittal plane, sEMG surface EMG signal, max(ABS) maximum ABS during a stride, ${sEMG|}_{t=t_{\max\left(ABS\right)}}$ value of surface EMG at that instant.	[94]
Ratio of height of first peak	EMG	EMG: shank (tibialis anterior)	Height of peak at origin in autocorrelation of filtered EMG signal, in a given window.	[38,110]
Lag of first peak (not at origin)	EMG	EMG: shank (tibialis anterior)	Autocorrelation of filtered EMG signal, in a given window.	[38,110]
Pearson’s correlation coefficient (PCC)	Acc, Gyro, FSR	Shanks, thighs, waist, FSR: under feet	Similarity between two signals, with *n* sample points, $x_{i}$, $y_{i}$, *i^th^* value of *x* and *y* signals; means $\bar{x}$, $\bar{y}$ $$PCC=\frac{\sum_{i=1}^{n}\left(x_{i}-\bar{x}\right)\left(y_{i}-\bar{y}\right)}{\sqrt{\sum_{i=1}^{n}{\left(x_{i}-\bar{x}\right)}^{2}}\sqrt{\sum_{i=1}^{n}{\left(y_{i}-\bar{y}\right)}^{2}}}$$ Calculated between acceleration axes or between FSR force of a step compared to template “normal” step.	[37,50,57,74,75,81]
Ground reaction force	FSR	Under heel, ball of foot	Sum of forces from all force sensing resistors (FSR) under a foot.	[37]
Shank displacement	Acc, Gyro	Shanks	Shank displacement (m) calculated from vertical acceleration and pitch angular velocity [111].	[50]
Change of the shank transversal orientation	Gyro	Shanks	Rotation angle in transversal plane, calculated as integral of angular velocity data about vertical axis, for each limb and each stride.	[50]
Auto regression coefficient	Acc	Waist	Four auto-regression coefficients obtained by Bourg method from acceleration in all 3 axes [112].	[57,74,81]
Entropy	Acc, Gyro, EEG	Acc: ankle, pants pocket, waist, wrists, chest, thigh Gyro: chest, waist, low back EEG: head	Shannon’s entropy: $H\left(x\right)=-\sum_{i=1}^{n}P\left(x_{i}\right)log_{2}P\left(x_{i}\right)$ where discrete variable *x* contains *n* values, *P* is probability (often defined from histogram), calculated from each axis of acceleration or angular velocity in time and frequency domains, or filtered EEG voltage from multiple scalp locations.	[39,42,43,45,54,64,66,87,96]
Direct transfer function	EEG	Head	Application of coherence directionality in multi-variate time series [113]. Signals from motor control regions: O1-T4 (visual), P4-T3 (sensorimotor affordance), Cz-FCz (motor execution) and Fz-FCz (motor planning). Data filtered band-pass (0.5–60 Hz), band-stop (50 Hz), then normalized with a z-transformation.	[99]
ICAIndependent component analysis	EEG	Head	Independent component analysis, used to maximize separation between signal components. Signals from motor control regions: O1-T4 (visual), P4-T3 (sensorimotor affordance), Cz-FCz (motor execution) and Fz-FCz (motor planning). Data filtered bandpass (0.5–60 Hz), band-stop (50 Hz), then normalized with a z-transformation.	[99]
Raw FFT	Acc, gyro, Goniometer (G)	Waist, shank, G: knee joint	The output signal from FFT. Calculated using acceleration, derivative of knee angle or angular velocity in the sagittal plane, in given window.	[35,50,64]
PSD bands	Acc, EEG, Goniometer (G), Telemeters (T)	Heels, shank and thighs, knee, shanks. G: knee T: between shanks	Specific frequency bands of power spectral distribution (PSD), generated by FFT, short-time FFT (SFFT), Z-transformation, or other method to convert time domain signal into frequency domain. Calculated from each acceleration and angular velocity axis, knee angular rotation, telemeter voltage, or filtered EEG voltages.	[30,55,66,80]
Ratio of peak frequencies	Goniometer	Knee angle	Computed from FFT of derivative of knee angle. Ratio of highest amplitude in 3–8 Hz divided by highest amplitude in 0.3–3 Hz.	[35]
Power in frequency domain	Acc	Ankle, shanks, thighs, waist, chest, wrists	Area under curve of power spectral density plot, between specific bands. From acceleration 3D vector magnitude or individual axes. Also, ratio of specific bands (similar to FI) [86].	[24,25,31,32,33,34,40,42,52,54,58,75,80,86]
Freeze index (FI)	IMU (Acc), Goniometer (G), Telemeter (T)	Acc: Various locations and sensor orientations, G: knees, T: between shanks	Ratio of signal power in freeze band (3–8 Hz) and locomotion band (0–3 Hz) [29] $FI=\frac{Area\;under\;the\;PSD\;curve\;in\;freeze\;band}{Area\;under\;the\;PSD\;curve\;in\;locomotion\;band}$ Calculated from acceleration and angular velocity axes, 3D vector magnitude, knee angular rotation or telemeter voltage.	[23,24,25,29,31,32,33,34,42,44,49,53,54,64,65,69,71,77,78,80,86,88,89,98]
Multi-channel FI ($FI_{MC}$)	Acc	Foot, shank, thigh, lower back/hip	Ratio of powers $P_{H}$ to $P_{L}$ (i.e., freeze and locomotor bands) that are summations of acceleration signal powers over *N* channels, where Matrix *X* of size *N × M* represents an *N*-channel recording session with *M* regularly spaced time samples $$FI_{MC}=\frac{P_{H}}{P_{L}}$$ $$P_{H}=\frac{1}{2f_{s}}\overset{N}{\underset{n=1}{\sum }}\left[\overset{H_{2}}{\underset{i=H_{1}+1}{\sum }}\left[P_{XX_{n}}\left(i\right)\right]+\overset{H_{2}-1}{\underset{i=H_{1}}{\sum }}\left[P_{XX_{n}}\left(i\right)\right]\right]$$ $$P_{L}=\frac{1}{2f_{s}}\overset{N}{\underset{n=1}{\sum }}\left[\overset{H_{1}}{\underset{i=L+1}{\sum }}\left[P_{XX_{n}}\left(i\right)\right]+\overset{H_{1}-1}{\underset{i=L}{\sum }}\left[P_{XX_{n}}\left(i\right)\right]\right]$$ where *N* is number of inputs, $f_{s}$ sampling frequency, $P_{XX}$, power spectrum of signal *x*, $H_{1}=\frac{3N_{FFT}}{f_{s}}$, $H_{2}=\frac{8N_{FFT}}{f_{s}}$, $L=\frac{0.5N_{FFT}}{f_{s}}$	[67]
K freeze index ($FI_{K}$)	Acc	Foot, shank, thigh, lower back/hip	Freeze index from each acceleration signal axis, spectral analysis using the Koopman operator [114]. Koopman eigenvalues and eigenfunctions are considered frequencies (*λ*) and power (*K*(*λ*)) [115]. $$FI_{K}=\frac{\sum_{\lambda =H_{1}+1}^{H_{2}}K\left(\lambda \right)}{\sum_{\lambda =L+1}^{H_{1}}K\left(\lambda \right)}$$ where $L=0.5\left(2\pi \right),\;H_{1}=3\left(2\pi \right),\;H_{2}=8\left(2\pi \right)$	[67]
Total power	Acc	Lower back, thigh, shank	$TTP=\sum_{j=1}^{M}P_{j}$ where *P* is the power spectrum of the acceleration signal for a window of length *M* [116,117]. Calculated for 3 axes	[86]
Mean power	Acc	Lower back, thigh, shank	$MNP=\frac{1}{M}\sum_{j=1}^{M}P_{j}$ where *P* is power spectrum of acceleration signal for window of length *M* [116,117]. Calculated for 3 axes.	[86]
Energy Derivative ratio (EDR)	Acc	Lateral waist	Derivative of vertical acceleration energy in 3–8 Hz band divided by derivative of energy in 0.5–3 Hz band.	[49,77]
Median frequency	Acc	Lower back, thigh, shank	$MDF=\frac{1}{2}\sum_{j=1}^{M}P_{j}$ where *P* is the power spectrum of acceleration signal for a window of length *M* [116,117]. Calculated for 3 axes.	[86]
Peak frequency	Acc	Lower back, thigh, shank	$PKF=max\left(P_{j}\right),\;j=1,\;\ldots ,\;M$ where *P* is power spectrum of acceleration signal for a window of length *M* [116,117]. Calculated for 3 axes.	[86]
Peak amplitude, Frequency of peak amplitude	Acc	Waist, thighs, shanks	Maximum value in frequency domain and corresponding frequency bin. Calculated for [0.5–3 Hz] band and [3–8 Hz] band. In [41] relative acceleration signal is used, defined in [105].	[41,64]
Higher harmonics	Acc	Waist, shanks	3 frequency bins with highest peaks. Calculated for all acceleration axes.	[57,64,74]
Frequency standard deviation	Acc	Waist, thighs, shanks, FSR in-shoe insoles	Standard deviation of signal in specific frequency bands, e.g., 0.1–0.68 Hz, 0.68–3 Hz, 3–8 Hz, 8–20 Hz, 0.1–8 Hz. Calculated for 3 axes.	[57,64,74]
Spectral density centre of mass (COM)	Acc, EEG, Goniometer (G), Telemeters (T)	Acc: Waist, thigh, shank, foot, EEG: Head, G: knee, T: between shanks	*x*(*n*), is amplitude of bin *n*, and *f*(*n*) is frequency of bin *n*: $COM=\frac{\sum_{n=0}^{N-1}f\left(n\right)x\left(n\right)}{\sum_{n=0}^{N-1}x\left(n\right)}$ Calculated from 3 axis acceleration signal, filtered EEG voltage calculated within specific frequency bands, knee angular rotation or telemeter voltage.	[57,66,74,81,86,96]
1^st^ 2^nd^ 3^rd^ spectral moments	Acc	Lower back, thigh, shank	$SM1=\sum_{j=1}^{M}f_{j}P_{j},\;\;SM2=\sum_{j=1}^{M}f_{j}^{2}P_{j},\;\;SM3=\sum_{j=1}^{M}f_{j}^{3}P_{j}$ where *P* is power spectrum of acceleration signal for window of length *M* [116,117]. Calculated for 3 axes.	[86]
Spectral coherence	Acc, EEG	Lower back, thigh, shank, EEG: head	Calculated from 3D acceleration or filtered EEG data using Welch method [118] $$C_{xy}\left(\omega \right)=\frac{P_{xy}\left(\omega \right)}{\sqrt{P_{xx}\left(\omega \right)\;P_{yy}\left(\omega \right)}}$$ where *ω* is frequency, $P_{xx}\left(\omega \right)$ is power spectrum of signal *x*, $P_{yy}\left(\omega \right)$ is power spectrum of signal *y*, and $P_{xy}\left(\omega \right)$ is cross-power spectrum for signals *x* and *y*. Also used with wavelet power spectrum in [96]. EEG signal from 4 locations: O1-visual, P4-sensorimotor affordance, Cz-motor execution, and Fz-motor planning. Filtered bandpass (0.5–60 Hz).	[20,67,69,96]
Max amplitude and number of peaks of spectral coherence	Acc	Foot, shank, thigh, lower back/hip	Maximum amplitude and number of peaks of spectral coherence feature [20].	[67]
Discrete wavelet transform (DWT)	Acc, EMG	Lower back, thigh, shank, EMG: quadriceps	Discrete wavelet transform, Decomposition coefficients (approximate and detail coefficients) used as features. Calculated from the acceleration 3D vector magnitude each axis individually, or the raw EMG signal.	[51,56,71,79]
Select bands of the CWT	Acc	Lower back, thigh, shank	Continuous wavelet transform in specific ranges (0.5–3 Hz, 3–8 Hz), also ratio of signal in 0.5–3 Hz band divided by signal in 0.3–8 Hz. Calculated for 3 axes.	[63]
Ratio of peak amplitude in wavelet transform bands	Goniometer,	Knee, derivative of knee angle	Sinusoidal wavelet transform used to calculate ratio of peak amplitude in 3–8 Hz band divided by peak in 0.5–3 Hz band.	[35]
S-transform, amplitude	EEG	Head	Maximum amplitude in theta (4–8 Hz), alpha (8–13 Hz), low beta (lβ, 13–21 Hz) and high beta (hβ, 21–38 Hz) bands. Total amplitude across all bands were extracted for a specific time. Electrodes placed: F3, F4, FC1, FC2, C3, C4, CP1, CP2, CZ, P3, P4, PZ and O1, O2, OZ (F = frontal, C = central, P = parietal, O = occipital and Z = midline). Data filtered band-pass filter (0.5–40 Hz), normalized with z-transformation.	[76]
Energy (frequency domain)	Acc, Gyro	Foot, shank, thigh, forearm, waist, chest, ankle	Summation of squared absolute value of signal, where *f*(*h*) is discrete signal in frequency domain, with frequency bins *h* = 1 to *H,* and *E* is signal energy $E=\overset{H}{\underset{h=1}{\sum }}{\left|f\left(h\right)\right|}^{2}$ Calculated from 3 axis acceleration or angular velocity signal.	[42,49,50,54,75,77,119]
Min, max amplitude of FFT and DWT	Acc	Shank, thigh, low back	Minimum and maximum values of energy of frequency domain signal, for both FFT and DWT approximation and detail coefficients, as in [92,93]. Calculated from 3 axis acceleration signal.	[83,91,92,93]
Cross-correlation	EEG	Head	$R_{xy}\left(k\right)=E\left[x\left(n\right)y\left(n+k\right)\right]$ where x(n), and y(n+k) are two signals and k is the number of time units that signal y(n) lags x(n), and E[·] is expectation operator. EEG signal from O1-visual, P4-sensorimotor affordance, Cz-motor execution, and Fz-motor planning. Filtered band-pass (0.5–60 Hz).	[96]
Cross power spectral density (CPSD)	EEG	Head	Cross power spectral density [120] $P_{xy}\left(f\right)=\sum_{k=-\infty }^{\infty }R_{xy}\left(k\right)e^{-j2\pi fkT}$ where *R* is cross correlation function. EEG signal from 4 locations: O1-visual, P4-sensorimotor affordance, Cz-motor execution, and Fz-motor planning. Filtered band-pass (0.5–60 Hz).	[96]
Weighted Phase Lag Index (WPLI)	EEG	Head	Weighted phase lag index [121]. EEG signal from 4 locations: O1-visual, P4-sensorimotor affordance, Cz-motor execution, and Fz-motor planning. Filtered band-pass (0.5–60 Hz).	[96]
Wavelet cross spectrum	EEG	Head	The wavelet cross spectrum $WCS_{i}\left(s\right)$, defined as $$WCS_{xyi}\left(s\right)=S\left(W_{xi}\left(s\right)W_{yi}^{*T}\left(s\right)\right)$$ where x and y are two time series, i time shift index, s scale, S a smoothing operator, and $W_{xi}$ and $W_{yi}$ the wavelet transform coefficients. EEG signal from 4 locations: O1-visual, P4-sensorimotor affordance, Cz-motor execution, and Fz-motor planning. Filtered band-pass (0.5–60 Hz).	[96]
Phase locking value	EEG	Head	Phase locking value [122] $PLV_{t}=\frac{1}{N}\left|\sum_{n=1}^{N}Ne^{j\theta \left(t,n\right)}\right|$ where θ(t,n) is phase difference between signals which can be derived from the angles of their wavelet coefficients. EEG signal from 4 locations: O1-visual, P4-sensorimotor affordance, Cz-motor execution, and Fz-motor planning. Filtered band-pass (0.5–60 Hz).	[96]

**Table 3:** Top machine-learning methods from studies that compared different machine-learning classifiers for FOG detection using wearable sensors.

Machine-Learning Methods Tested	Best Method	Second Best	Third Best	Source
Random forests, decision trees, naive Bayes, *k*-nearest neighbor (KNN-l) (KNN-2), multilayer perceptron NN, boosting (AdaBoost) and bagging with pruned decision trees.	AdaBoosted decision tree (1 s window)	Random forest (1 s window)	Bagging with decision tree (1 s window)	[42]
Naïve Bayes, random forest, decision trees, random tree.	Random forest (1 s window)	Decision tree (1 s window)	Random tree (1 s window)	[43]
*k*-nearest neighbor, random forest, logistic regression, naïve Bayes, multilayer perceptron NN, support vector machine.	Support vector machine (1.6 s window)	Random forest (1.6 s window)	Multilayer perceptron NN (1.6 s window)	[57,81]
Convolutional NN, decision trees with bagging, Adaboosting, logitBoost, RUSBoost, robustBoost) support vector machine.	Convolutional NN (2.56 s window)	Support vector machine (2.56 s window)	RUSBoost (2.56 s window)	[85]
